# Inhibition of Cardiac Hypertrophy Effects in D-Galactose-Induced Senescent Hearts by* Alpinate Oxyphyllae Fructus* Treatment

**DOI:** 10.1155/2017/2624384

**Published:** 2017-04-05

**Authors:** Yung-Ming Chang, Hen-Hong Chang, Hung-Jen Lin, Chin-Chuan Tsai, Chuan-Te Tsai, Hsin-Nung Chang, Shu-Luan Lin, Vijaya PadmaViswanadha, Ray-Jade Chen, Chih-Yang Huang

**Affiliations:** ^1^The School of Chinese Medicine for Post-Baccalaureate, I-Shou University, Kaohsiung 840, Taiwan; ^2^Chinese Medicine Department, E-Da Hospital, Kaohsiung 824, Taiwan; ^3^1PT Biotechnology Co., Ltd., Taichung 433, Taiwan; ^4^Research Center for Chinese Medicine & Acupuncture, China Medical University, Taichung 404, Taiwan; ^5^Department of Chinese Medicine, China Medical University Hospital, Taichung 404, Taiwan; ^6^School of Post-Baccalaureate Chinese Medicine, College of Chinese Medicine, China Medical University, Taichung 404, Taiwan; ^7^Graduate Institute of Basic Medical Science, China Medical University, Taichung 404, Taiwan; ^8^1PT Lukang Chinese Medicine Clinics, Changhua 505, Taiwan; ^9^Department of Biotechnology, Bharathiar University, Coimbatore 641046, India; ^10^Department of Surgery, School of Medicine, College of Medicine, Taipei Medical University, Taipei 110, Taiwan; ^11^School of Chinese Medicine, China Medical University, Taichung 404, Taiwan; ^12^Department of Health and Nutrition Biotechnology, Asia University, Taichung 413, Taiwan

## Abstract

Aging is a complex physiological phenomenon accelerated by ROS accumulation, with multisystem decline and increasing vulnerability to degenerative diseases and death. Cardiac hypertrophy is a key pathophysiological component that accompanies the aging process. Alpinate Oxyphyllae Fructus (*Alpinia oxyphylla* MIQ, AOF) is a traditional Chinese medicine, which provides cardioprotective activity against aging, hypertension, and cerebrovascular disorders. In this study, we found the protective effect of AOF against cardiac hypertrophy in D-galactose-induced aging rat model. The results showed that treating rats with D-galactose resulted in pathological hypertrophy as evident from the morphology change, increased left ventricular weight/whole heart weight, and expression of hypertrophy-related markers (MYH7 and BNP). Both concentric and eccentric cardiac hypertrophy signaling proteins were upregulated in aging rat model. However, these pathological changes were significantly improved in AOF treated group (AM and AH) in a dose-dependent manner. AOF negatively modulated D-galactose-induced cardiac hypertrophy signaling mechanism to attenuate ventricular hypertrophy. These enhanced cardioprotective activities following oral administration of AOF reflect the potential use of AOF for antiaging treatments.

## 1. Introduction

Cardiac hypertrophy is an adaptive cellular response associated with increased biomechanical stress, such as hypertension, hypertrophic cardiomyopathy, myocardial infarction, and vascular heart disease [1]. It is well known that cardiac hypertrophy is an important compensation mechanism in response to pathological stress; the heart adapts to pressure and volume overload by undergoing hypertrophic enlargement without cell proliferation. Even though the initial compensation response may be beneficial, continued hypertrophy eventually leads to heart failure and ultimately death [2–4]. Pressure and volume overload usually elicits concentric hypertrophy and eccentric hypertrophy, respectively [5, 6]. Cardiac hypertrophies usually accompany the increscent of fetal genes expression, including atrial natriuretic peptide (ANP) and brain natriuretic peptide (BNP); both are elevated in cardiac hypertrophy and have been characterized as a feature of hypertrophy in all mammalian species [7–9]. Cytokine Interleukin-6 (IL6), a potent hypertrophic effecter, triggers glycoprotein 130 (gp130) dimerizing and activating downstream hypertrophic signaling [10, 11]. Previous study indicated that IL6-related pathways play a crucial role in eccentric cardiac hypertrophy, including IL6-related MEK5-ERK5 signaling and JAK2-STAT3 signaling. These hypertrophy-related molecular signals were activated simultaneously with IL6 expression and lead to morphological changes [12, 13]. Recently, reports showed that the hypoxia marker, BNIP3 (Bcl-2/adenovirus E1B nineteen-kDa interacting protein 3), induced concentric cardiac hypertrophy by targeting the mitochondria and endoplasmic reticulum (ER) activating the calcium-dependent signaling, calcineurin/NFAT3 [13, 14]. Another hypertrophic mediator, p38 MAPK, was found to mediate concentric cardiac hypertrophy by phosphorylated GATA4 to further expressed hypertrophic response genes such as ANP and BNP [13, 15–17].

Mitochondria are generally considered the major source of reactive oxygen species (ROS) production [18, 19]; other potential sources of ROS include NADPH oxidase, xanthine oxidase, and uncoupled nitric oxide synthase (NOS) [20–22]. Accumulating evidence demonstrates that oxidative stress impaired antioxidant defense due to ROS overproduction and have been implicated in the development and subsequent progression of related heart diseases especially cardiac hypertrophy [23–26]. ROS production increase has been shown to be involved in the hypertrophy of isolated cardiomyocytes induced by angiotensin II (AngII), tumor necrosis factor-a (TNFa), cyclic stretch, or a-adrenergic agonists [27–29]. Additionally, NADPH oxidase 4 (Nox4), a major source of oxidative stress in the failing heart which has been identified, induces cardiac hypertrophy through activating Akt/mTOR and NF*κ*B signaling [30, 31]. It has also been reported that Nox4 directly mediates mitochondrial dysfunction, oxidative stress, and myocardial cell death during pressure overload-induced cardiac hypertrophy [31, 32].

D-Galactose (D-gal) is a reducing sugar that has been widely used in age-related oxidative damage and aging pharmacology research [33–35]. D-Gal generates ROS during its metabolism in vivo by reacting readily with the free amines in proteins, lipids, and nucleic acids to form advanced glycation end products (AGEs) [36, 37]. Growing evidence suggests that AGEs are interacting receptors for AGE (RAGE) in many cell types and induce downstream NF-*κ*B and other signaling pathways eventually lead to ROS generation and accelerate the aging process [38–41].

Alpinate Oxyphyllae Fructus (Alpinia oxyphylla MIQ, AOF) is one of the important traditional Chinese medicines which has antiaging and sexual-reinforcing activity [42]. According to the Chinese pharmacopoeia, AOF has been widely used for treating gastralgia, diarrhea, ulceration, antitumor, hypertension accompanying symptoms, and cerebrovascular disorders [42–46]. An increasing number of evidences indicate that AOF extracts exhibit cardioprotective and neuroprotective activity against oxidative stress-induced apoptosis [44, 47]. Subsequent studies demonstrated that AOF extracts protect against Ang II induced cardiac apoptosis in H9c2 cardiomyoblast cell [48]. We here further investigate whether AOF ameliorate the ROS-induced aging heart problem and related signaling paths and mechanisms.

## 2. Materials and Methods

### 2.1. AOF Extraction

Fragmented Alpinate Oxyphyllae Fructus (*Alpinia oxyphylla* MIQ, AOF) was obtained from Shin-Long Pharmaceutical Company (Taichung, Taiwan).

The AOF fragment (150 g) was extracted by boiling in 0.6 L of boiling water for 2 h. The water extract was filtered by gravity-flow procedure at reduced pressure for convenience and then stored at 4°C. The water extract was spray dried to produce a powdered extract. The product yield percentage was 7.4%.

### 2.2. Animals and Experimental Design

Thirty-four male 8-week-old Sprague-Dawley rats weighing 220 ± 20 g were provided by the National Institutes of Health (NIH) colony and cared for at the Comparative Biology facility at Texas A&M University in accordance with NIH and ULACC (University Laboratory Animal Care Committee) standards. Rats were maintained on standard laboratory conditions of temperature (23 ± 2°C) and a 12 : 12 h light-dark cycle with water and rat chow available ad libitum for the duration of the study. After 2 weeks of acclimatization, the rats were randomly divided into 5 groups. One was normal control group (NC). The other four were induced aging groups injected with D-galactose (150 mg kg/day for 8 weeks); in induced aging groups, the three AOF groups were orally administered with AOF of 50 (AOF low, AL), 100 (AOF medium, AM), and 150 (AOF high, AH) mg/kg/day, respectively; the normal control group were given the same volume of control solution. After the animals were sacrificed, the heart tissue was immediately collected and stored at −80°C until further use.

### 2.3. Cardiac Characteristics

The whole hearts of animals were weighed after being excised and cleaned with PBS. The left ventricle tissues were isolated and weighed. The tibia length was measured by the electronic digital Vernier caliper to adjust the whole heart weight. The ratios of the whole heart weight to the tibia length and the left ventricle weight to tibia length were calculated.

### 2.4. Echocardiography

Transthoracic echocardiographic images of rats were performed using Hewlett-Packard Sonos 5500 ultrasound machine with a 15 MHz linear-array transducer. Left ventricular M-mode measurements at the level of the papillary muscles include interventricular septal thickness at end-diastole (IVSD), left ventricular internal dimension at end-diastole (LVIDd), left ventricular posterior wall thickness at end-diastole (LVPWd), and left ventricular internal dimension at end-systole (LVIDs).

### 2.5. Tissue Extraction

The left ventricle tissues were isolated and washed 3 times in PBS buffer and then weighed. Approximately 0.1 g tissue was added by 1 mL lysis buffer (0.1% SDS, 0.5% Na-deoxycholate, 1% NP-40, 2 mM EDTA, 50 mM TrisHCL, 50 mM NaF, and 150 mM NaCl) into the mixture. The tissue was homogenized for 20 min and centrifuged at 1,200 rpm at 4°C. After stirring and centrifugation at 12,500 rpm, a clean upper layer suspension was extracted. The homogenization was repeated, and a clean upper layer suspension was extracted.

## 3. Senescence-Associated *β*-Galactosidase Staining

The tissue sections were fixed in 0.2% glutaraldehyde and 2% formaldehyde at room temperature for 15 min. Sections were washed three times in PBS and incubated in freshly senescence-associated *β*-galactosidase (SA-*β*-gal) staining solution (1 mg/mL X-gal, 40 mM citric acid/sodium phosphate (pH 6.0), 5 mM potassium ferricyanide, 5 mM potassium ferrocyanide, 150 mM NaCl, and 2 mM MgCl_2_) for 14 h at 37°C without CO_2_. SA-*β*-gal staining was visualized under an Olympus (Tokyo, Japan) microscope.

### 3.1. Hematoxylin-Eosin (H&E) Staining

The tissue sections were dyed using hematoxylin and eosin (H&E). After the hearts were excised, tissue sections were stained using hematoxylin and eosin (H&E). Sections were dewaxed by immersion in xylene and dyed with hematoxylin for 5 min; the sections were washed three times in double-distilled water (DDW) and soaked in 85% alcohol for 2 min. The sections were dyed with eosin for 5 min and dehydrated through graded alcohols (100%, 95% and 75%). Finally, the sections were soaked in 100% alcohol for 5 min and two times in xylene for 1 min. The sections were then sorted and photographically analyzed using a microscope (Olympus Microscope).

### 3.2. Electrophoresis and Western Blot

Cardiac tissues extract protein concentration was determined by the Lowry protein assay. Tissue protein samples (40 *μ*g/lane) and 5x loading dye were mixed and placed on 95°C for 5 min and then separated on sodium dodecyl sulfate polyacrylamide gel electrophoresis (SDS-PAGE) with a constant voltage of 70 V. The upper SDS-PAGE layer was a 5% stacking gel, and the bottom layer was 8 or 10 or 12% separating gel.

Electrophoresed proteins were transferred to a polyvinylidene difluoride (PVDF) membrane (0.45 *µ*m pore size, Millipore, Bedford, MA, USA) with vertical electrophoresis system (Bio-Rad Laboratories Inc., Berkeley, CA, USA). The PVDF membranes were incubated in 5% fat-free milk in Tris-buffered saline (TBS) and shaken for 1 h at RT. After shaking, the PVDF membranes were incubated with the primary antibody overnight at 4°C. The primary antibody was diluted to 1 : 1000 in antibody binding buffer. The immunoblots were washed with TBS buffer 3 times for 10 min each and then incubated with the secondary antibody solution containing goat anti-mouse IgG-HRP, goat anti-rabbit IgG-HRP, or donkey anti-goat IgG-HRP (Santa Cruz Biotechnology) for 1 hr at room temperature. The secondary antibody was diluted to 1 : 3000 in TBS buffer. The membrane was washed again with washing buffer. Finally, the membrane was colored with an enhanced chemiluminescence ECL Western blotting luminal reagent (Santa Cruz Biotechnology) and membrane data was collected using an LAS-4000 mini (GE Healthcare Life Sciences). The data were quantified using Image J.

### 3.3. Statistical Analysis

All experiments were repeated at least three times. One-way ANOVA was used for comparisons between multiple groups. Student's *t*-test was used to compare two differences groups. Con and IA (induced aging) served as negative control (normal rats) and positive control (aging rats) groups, respectively. *p* < 0.05 was considered significant.

## 4. Results

### 4.1. Body Weight and Cardiac Characteristics

Rat heart weight and cardiac functional parameters were analyzed to compare differences between groups. The whole heart weight (WHW), left ventricular weight (LVW), WHW/tibia, LVW/tibia, and the echocardiographic parameter LVPWd increased in the IA group when compared to the control group (*p* < 0.05). AOF treatment for 10 weeks significantly decreased the aging effects of the treated IA rats as compared with the IA group (*p* < 0.05), as shown in Table 1. These results indicated that the AOF treatment was effective in D-galactose induced aging rat model.

### 4.2. AOF Treatment Reduced Senescent Changes in Induced Aging Rat Hearts

**Figure 1:**
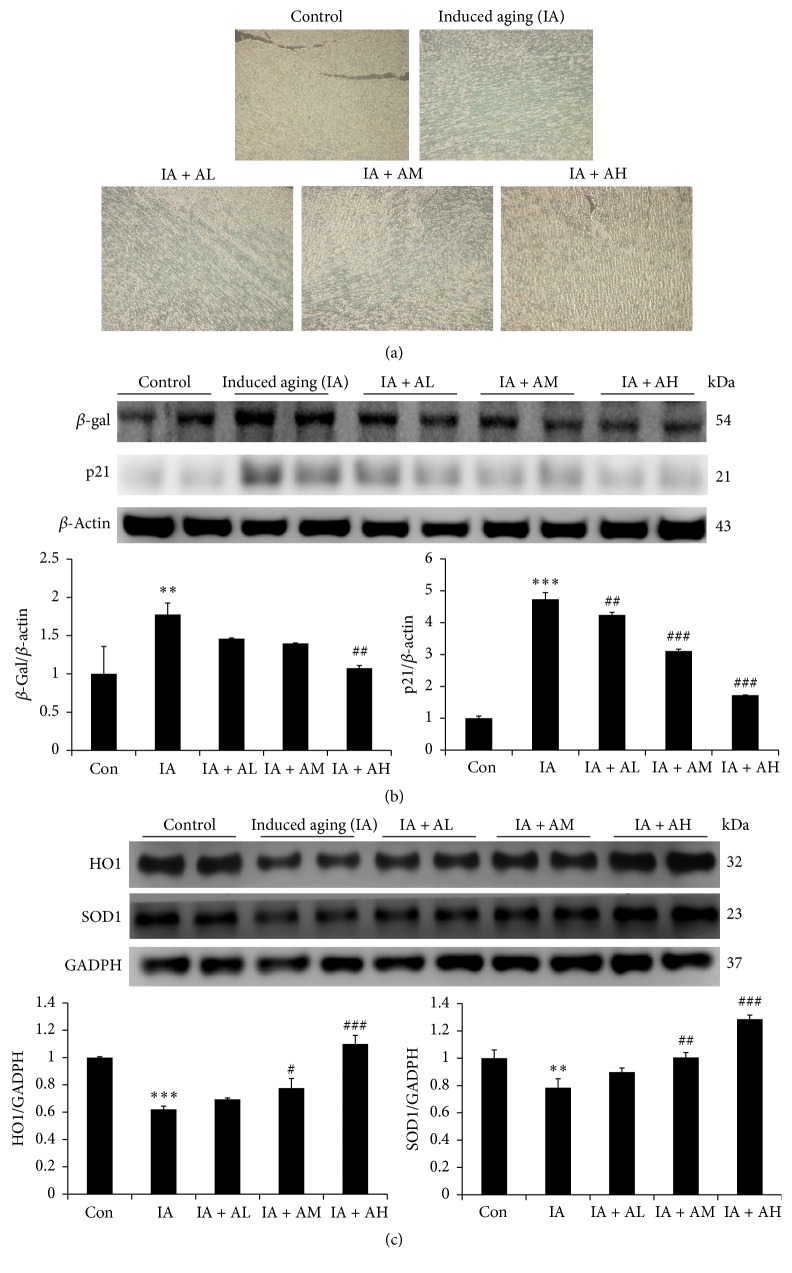
AOF treatment reduced senescent changes in aged rat hearts. (a) SA-b-gal staining results for cardiac tissues from study groups of rats. Blue precipitation in the cytoplasm was observed in the senescent cells. ×200 magnification. (b) Representative aging-related protein products extracted from the left ventricles of study rats in each group. (c) Representative antioxidants protein products extracted from the left ventricles of study rats in each group, control rats; IA, induced aging rats; AL, AOF low; AM, AOF medium; AH, AOF high, were measured using Western blotting. *β*-Actin and GADPH were used as an internal control.   ^*∗∗*^*p* < 0.01 and ^*∗∗∗*^*p* < 0.001 compared with the control group; ^#^*p* < 0.05, ^##^*p* < 0.01, and ^###^*p* < 0.001 compared with the induced aging. *n* = three independent experiments for each data point.

We examined the expression of SA-*β*-gal, a well-defined in vivo senescence marker [49–51]. The intensity of SA-*β*-gal staining in induced aging (IA) rat hearts was significant increase in the percentage of SA-*β*-gal accumulation compared to the control group (*p* < 0.05; Figure 1(a)). However, in the IA + AOF treatment group (AL, AM, and AH), the percentage of SA-*β*-gal accumulation was significantly reduced (*p* < 0.05; Figure 1). Furthermore, the senescence biomarkers SA-*β*-gal and p21 were significantly increased in the IA group compared with those in control group but were seen to be regulated in the groups treated with AOF (Figure 1(b)).

D-Galactose treatment remarkably decreased the protein levels of HO1 and SOD1, but AOF treatment ameliorated the effect in a dose-dependent manner (Figure 1(c)). These findings suggest that AOF treatment can protect cardiac cell against senescence.

### 4.3. Cardiac Histopathological Changes Analyses of Cardiac Tissue

**Figure 2:**
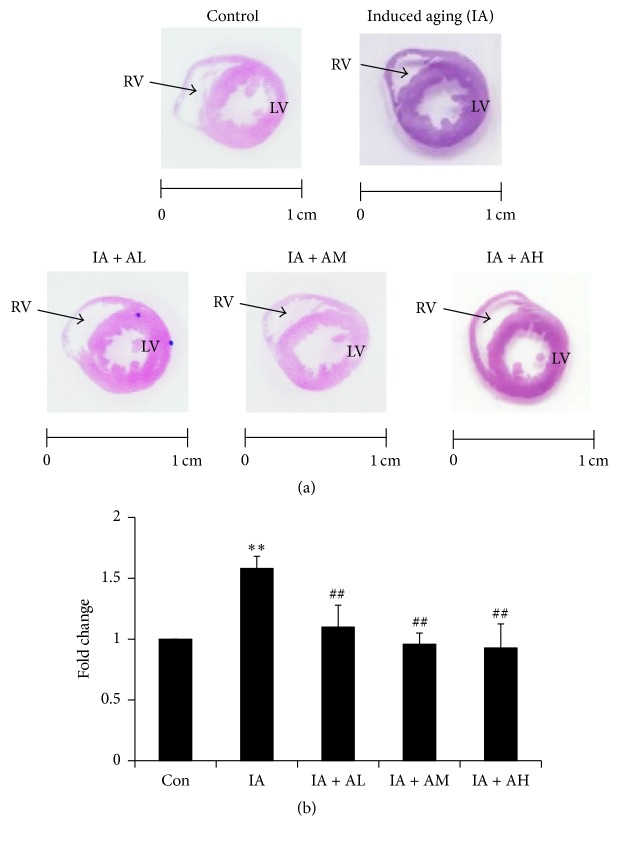
Hematoxylin and eosin staining (H&E stain). Cardiac tissue sections stained with hematoxylin and eosin. (a) The myocardial architecture images were magnified ×10. The scale bar is 1 cm. RV: right ventricle and LV: left ventricle. (b) Quantization of cardiac ventricular area (^*∗∗*^*p* < 0.01 compared with the control group; ^##^*p* < 0.01 compared with the IA group). *n* = three independent experiments for each data point.

In order to investigate whether AOF treatment improves cardiac architecture, we performed histopathological tomography analyses of whole heart tissues with hematoxylin-eosin staining (Figure 2). H&E stained sections of the IA group showed increased LV wall thickening compared to that of the control group but this abnormality was reduced in AOF treatment groups (AL, AM, and AH) (Figure 2).

### 4.4. AOF Treatment Suppressed Cardiac Hypertrophy in D-Galactose-Induced Senescence Effects in SD Rat Hearts

**Figure 3:**
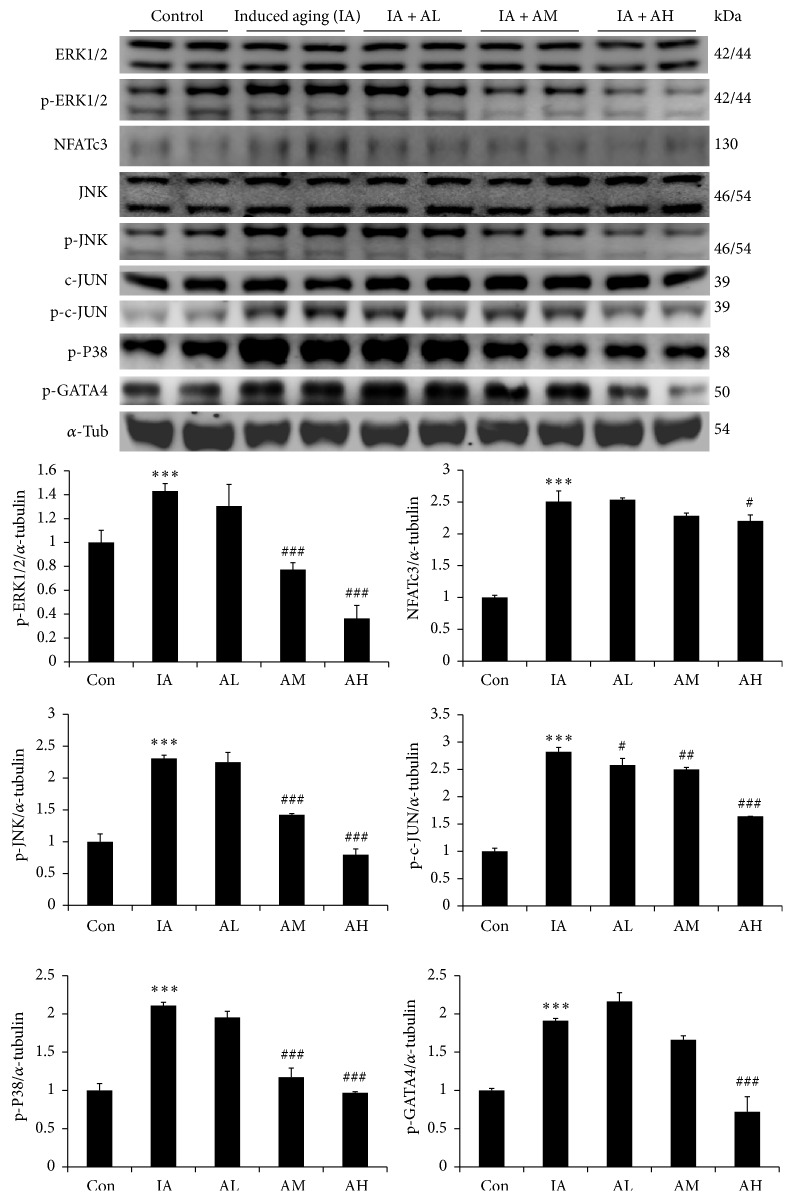
Representative concentric hypertrophy protein products extracted from the left ventricles of study rats in each group, control rats; IA, induced aging rats; AL, AOF low; AM, AOF medium; AH, AOF high, were measured using Western blotting. *α*-Tubulin was used as an internal control.  ^*∗∗∗*^*p* < 0.001 compared with the control group; ^#^*p* < 0.05, ^##^*p* < 0.01, and ^###^*p* < 0.001 compared with the induced aging. *n* = three independent experiments for each data point.

**Figure 4:**
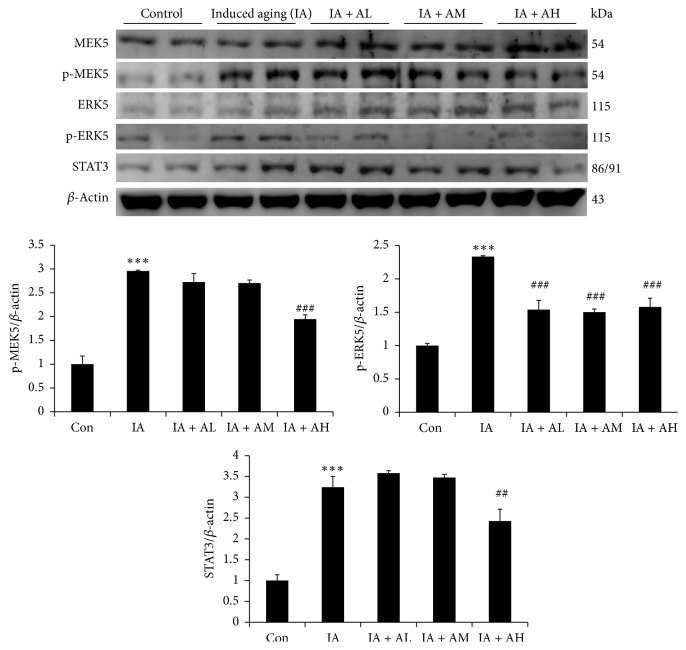
Representative eccentric hypertrophy protein products extracted from the left ventricles of study rats in each group, control rats; IA, induced aging rats; AL, AOF low; AM, AOF medium; AH, AOF high, were measured using Western blotting. *β*-Actin was used as an internal control.   ^*∗∗∗*^*p* < 0.001 compared with the control group; ^##^*p* < 0.01 and ^###^*p* < 0.001 compared with the induced aging. *n* = three independent experiments for each data point.

To further identify whether pathological hypertrophy signaling pathways were altered in AOF treated aging rats, the genes involved in pathological hypertrophy were examined by Western blotting. The data indicated that the concentric hypertrophy-related MAPKs such as p-ERK1/2, p-c-JUN, p-JNK, and p-p38 were significantly increased in the IA group compared with those in control group. Moreover, the pathological hypertrophy associated transcription factors such as NFATc3 and p-GATA4 were increased in the aging rats hearts (Figure 3). However, the p-ERK1/2, p-c-JUN, p-JNK, p-p38, NFATc3, and p-GATA4 in the AOF treatment groups (AM and AH) were significantly lower than those in the IA group (Figure 3). The eccentric pathological related protein p-MEK5, p-ERK5, and transcription factors STAT3 were also significantly increased in aging rats models. However, all these pathological changes were reversed by AOF in a dose dependent manner (Figure 4).

### 4.5. Dose-Dependent Effects of AOF on BNP, MYH6, and MYH7 Protein Levels

**Figure 5:**
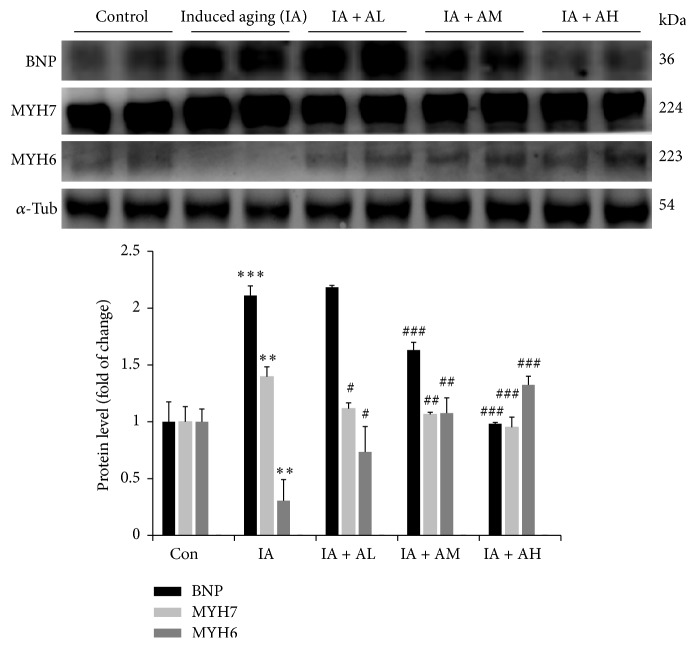
Dose-dependent effect of AOF on the hypertrophic protein expression levels from the left ventricles of study rats in each group. BNP, MYH6, and MYH7 protein levels were determined by Western blot analysis. *α*-Tubulin was used as an internal control. ^*∗∗*^*p* < 0.01 and ^*∗∗∗*^*p* < 0.001 compared with the control group; ^#^*p* < 0.05, ^##^*p* < 0.01, and ^###^*p* < 0.001 compared with the induced aging. *n* = three independent experiments for each data point.

After confirming the effects of AOF treatment on pathological hypertrophy associated signaling pathways, we investigated the physiological hypertrophy-related markers BNP, MYH6, and MYH7 [52–54] protein levels by Western blot analysis. Our results showed that D-galactose-induced aging effects caused a significant increase in the protein expression levels of both BNP and MYH7 by approximately twofold and 40%, respectively. However, MYH6 protein level was upregulated by AOF treatment in a dose-dependent manner to reach approximately 30% of its control value (Figure 5).

## 5. Discussion

D-Galactose induced oxidative stress in animal models to mimic natural aging has been wildly used in antiaging pharmacological studies [55]. Oxidative stress is increased due to abnormal metabolism such as glucose autoxidation and generation of AGE that results in the increase of reactive oxygen species (ROS) and eventually contributes to cardiac hypertrophy [56, 57]. It is well known that oxidative stress is generated via ROS and has been implicated in the development of cardiac hypertrophy [57, 58]. The aim of the present study was to investigate the cardiac protective role and the mechanism of AOF against pathological hypertrophy in D-galactose induced aging rats. Senescence staining and immunoblot show that D-galactose induces oxidative stress resulting in cardiac aging. Additionally, D-galactose treatment remarkably decreased the protein level of SOD1 and HO1 and AOF supplementation showed significant protection effect by elevating these antioxidants. Furthermore, whole heart weight, left ventricular weight, and left ventricular posterior wall thickness at end-diastole significantly increased in the aging groups. However, AOF treatments ameliorate age-related functional deterioration and promoted the hearts of IA group rats to maintain a relatively healthy physiological state. Therefore, AOF treatment potentially provides positive effects on cardiac cell morphology and cardiac contraction. AOF is a traditional Chinese medicine that exhibits cardioprotective activity against oxidative stress-induced apoptosis [44, 47]. Accumulating evidence demonstrates that AOF possesses diverse pharmacologic effects including antioxidant, anti-inflammatory, antitumor, and antiapoptosis [59–62]. Thus, we expected AOF may provide a recovery effect against cardiac hypertrophy in D-galactose-induced aged rat. We further examined the mechanism of aging-induced cardiomyocyte hypertrophy, which occurred through both concentric and eccentric hypertrophy pathways.

Previous studies indicate that ERK1/2 and JNK1/2 phosphorylate various substrates such as transcription factors GATA4 and NFATs, subsequently triggering the transcription of hypertrophy-related genes, and eventually result in cardiac hypertrophy [63, 64]. Our current results showed that the expression of cardiac pathological and concentric hypertrophy proteins ERK1/2/JNK, p-P38, and NFATc3 was markedly increased in rat hearts with D-galactose treatment. We further observed that the eccentric-related MEK5-ERK5 signaling and the transcription factor STAT3 protein were also upregulated in aging rats hearts. Notably, AOF treatment conferred protection against cardiac hypertrophy via downregulation of both concentric- and eccentric-related hypertrophy signaling pathway.

In mammals, the myosin heavy-chain isoforms MYH6 and MYH7 have been identified as cardiac motor proteins that are crucial determinants of contractile performance in cardiac muscle tissue [65, 66]. Emerging evidence demonstrated that MYH7 protein expression was significantly increased in cardiac hypertrophy and has been identified as hypertrophy-related marker [67]. Subsequent studies indicate that cardiac hypertrophy usually accompanies upregulation of BNP and MYH7 and/or downregulation of MYH6 expression levels [68]. In the present study, high BNP and MYH7 expressions were observed following D-galactose treatment in the IA groups. Treatment with AOF in IA rats significantly decreased the hypertrophy-related markers BNP and MYH7 in a dose-dependent manner in cardiomyocytes. Systematic survey on literatures shows that over the past decade at least 80 chemical constituents were reported to be identified from* A. oxyphylla* that include sesquiterpenes, flavonoids, diarylheptanoids, steroids, volatile oil, and their glycosides [62, 69–78]. Recently, Chen et al. [79] evaluated the abundance of nine secondary metabolites in* A. oxyphylla* capsular fruits, including flavonoids (e.g., tectochrysin, izalpinin, chrysin, apigenin-4′,7-dimethyl ether, and kaempferide), diarylheptanoids (e.g., yakuchinone A, yakuchinone B, and oxyphyllacinol), and sesquiterpenes (e.g., nootkatone). The nine secondary metabolites were differentially concentrated in seeds and fruit capsules. Furthermore, the optimized condition for the extraction and the content levels of nine compounds of different harvest time has been previously evaluated by Li et al. [80] and the remarkable differences in chemical markers at different harvest time by Li et al. [80]. Additionally, protocatechuic acid (PCA), one of the major active ingredients in the kernels of AOF, has been reported as an efficient cytoprotective agent against oxidative stress, neurotoxicity, and apoptosis [42–44, 46]. Recently, we reported that PCA effectively promoted RSC96 cell survival and proliferation by activating IGF-IR-PI3K-Akt signaling [81]. Moreover, PCA induced the migration and regeneration of RSC96 Schwann cells via ERK1/2, JNK1/2, and p38 MAPK pathways activation [82]. The protective effects associated with the chemical constituents of PCA correlate with the pharmacological activities of AOF as defined in the present study.

In conclusion, our findings suggest the beneficial effects of AOF on cardiac hypertrophy. The mechanism by which AOF protects against cardiac hypertrophy may be partly via blockade of both concentric and eccentric signaling pathways. D-Galactose-induced pathological hypertrophy associated proteins as well as expression levels of BNP and MYH7 could attenuate by AOF treatment. Therefore, AOF might be a potential candidate for the treatment of aging-induced cardiovascular hypertrophy and heart failure.

## Figures and Tables

**Table 1:** Heart weight and echocardiographic indices.

	Control	Induced aging (IA)	IA + AL	IA + AM	IA + AH
	*n* = 4	*n* = 4	*n* = 4	*n* = 4	*n* = 4
Body weight (g)	456 ± 31.05	462 ± 32.32	437 ± 61.02	444 ± 31.4	464 ± 15.6
WHW (g)	1.39 ± 0.06	1.55 ± 0.07^*∗*^	1.39 ± 0.10	1.37 ± 0.03^#^	1.40 ± 0.03^#^
LVW (g)	0.99 ± 0.06	1.11 ± 0.06^*∗*^	0.95 ± 0.11	0.96 ± 0.02^##^	0.97 ± 0.02^##^
WHW/tibia (10^2^)	3.10 ± 0.09	3.52 ± 0.2^*∗∗*^	3.35 ± 0.23^#^	3.25 ± 0.08	3.37 ± 0.10^*∗*^
LVW/tibia (10^2^)	2.20 ± 0.09	2.54 ± 0.13^*∗∗*^	2.28 ± 0.24	2.30 ± 0.05^#^	2.33 ± 0.08^#^
IVSD (mm)	1.37 ± 0.08	1.31 ± 0.12	1.43 ± 0.16	1.31 ± 0.12	1.38 ± 0.10
LVIDd (mm)	9.15 ± 0.62	9.67 ± 0.73	8.89 ± 0.34	9.20 ± 0.12	9.02 ± 0.50
LVPWd (mm)	1.32 ± 0.12	1.50 ± 0.14^*∗*^	1.35 ± 0.08	1.10 ± 0.09^###^	1.23 ± 0.07^##^
LVIDs (mm)	6.09 ± 0.75	6.17 ± 0.83	5.12 ± 0.27	5.99 ± 0.31	5.05 ± 0.25^*∗*^

Values are means ± SD; *n* = 4 at least in each group. AL, AOF low; AM, AOF medium; AH, AOF high; WHW, whole heart weight; LVW, left ventricular weight; WHW/tibia, whole heart weight normalized by tibia length; LVW/tibia, left ventricular weight normalized by tibia length; IVSD, interventricular septal thickness at end-diastole; LVIDd, left ventricular internal dimension at end-diastole; LVPWd, left ventricular posterior wall thickness at end-diastole; LVIDs, left ventricular internal dimension at end-systole. ^*∗*^*p* < 0.05 and ^*∗∗*^*p* < 0.01 compared to the control group; ^#^*p* < 0.05, ^##^*p* < 0.01, and ^###^*p* < 0.001 compared to the induced aging group, respectively.

## Abbreviations

BW: Body weight
WHW: Whole heart weight
LVW: Left ventricle weight
IVSD: Interventricular septal thickness at end-diastole
LVIDd: Left ventricular internal dimension at end-diastole
LVPWd: Left ventricular posterior wall thickness at end-diastole
LVIDs: Left ventricular internal dimension at end-systole.
